# Emotional intelligence training in health students: a pilot study

**DOI:** 10.3389/fspor.2025.1511851

**Published:** 2025-02-24

**Authors:** Manon Dugué, Olivier Sirost, Natacha Heutte, Fabrice Dosseville

**Affiliations:** ^1^ Sport Sciences Department, Université Rouen Normandie, Mont Saint Aignan, France; ^2^ EA3832Centre d’Etudes des Transformations des Activités Physiques et Sportives, Mont Saint Aignan, France; ^3^ Sport Sciences Department, Université Caen Normandie, Caen, France; ^4^ UR 7480 – Vertige Extrême VERTEX, Caen, France

**Keywords:** emotional competences, health student, physical activity, intervention, university

## Abstract

**Introduction:**

The aim of this study was to test the effectiveness of an emotional intelligence (EI) program based on the tripartite model, combining theory and practical physical activity for health training students.

**Method:**

This pilot study was conducted with 68 students between October 2020 and October 2021. One group received the EI training intervention (*n* = 34) combining 8 h of theory and 10 h of practical exercises with physical activities on different EC based on scientific literature. While the other group served as a control group (*n* = 34) and received an intervention on a topic other than emotional intelligence (i.e., sociological approach to the body) during 18 h (*n* = 34) Participants of both groups were required to complete the trait EI questionnaire in the pre and post-test.

**Results:**

Results showed a significant increase in emotion expression, emotion identification, stress management, emotional management, emotional abilities, sociability and emotional intelligence. No significant change was observed in the control group.

**Discussion:**

These findings suggest that EI can be improved with physical activities and open possibilities to include EI program in health training program.

## Introduction

The links between stress and health have been the subject of much research on health education. Indeed, in addition to academic stress (e.g., heavy workload, exams), students are confronted with additional stress during their internship (e.g., regular confrontation with the death or suffering of patients, difficult work conditions, time pressure (1–4). However, there are few training contents that teach them how to cope with situations perceived as stressful. It is essential to integrate tools that enable students to learn to cope with difficulties of the profession. In France, university training generally focuses on theoretical knowledge and tends to neglect development of psychological skills. Yet, different psychological determinants are negatively correlated with high stress: emotional competences (EC), coping strategies to deal with the problem or stress tolerance (5–8). Furthermore, given the stressful nature of health training, it seems appropriate to include EI development earlier and preventively in the training curriculum.

EC are defined as the ability of an individual to understand, use, identify, express and regulate their own and others’ emotions (9). Research on Emotional Intelligence (EI) is based on two conceptual models: the Ability model (10) and the Trait model (11, 12). Proponents of the Ability model view Emotional Intelligence (EI) as a cognitive skill responsible for processing emotion-related information (10). In contrast to the Ability model, the Trait approach views Emotional Intelligence (EI) as a characteristic associated with an individual's personality (11, 12). The tripartite model represents a third theoretical perspective on EI that is gaining traction, particularly in health-related contexts (9). This model is presented as a useful alternative to the traditional conceptualization of Emotional Intelligence (EI) as either a trait or an ability. These five EC could be broken down into three levels: knowledge, representing what the individual knows about emotional skills; ability, which is the application of this knowledge in an emotional situation when the individual is invited to do so in practical exercises; trait, representing how to deal with emotional situations (9). Furthermore, recent systematic reviews EC showed that they play an important role on well-being, physical health, performance and social relationships (13–15). Moreover, the implementation of intervention programs aimed to develop EC has been the subject of several studies (16, 17). However, the number of sessions and their duration differ widely between programs, and even more when it comes to the form and content of these interventions (e.g., conference, workshop, practical exercises…). In view of professional activity's requirements for the caregivers, we can then wonder about the most adequate tools used to enable them developing their emotional skills. For example, several studies show that regular physical activity is negatively correlated with stress and positively correlated with well-being, health, performances and social relationships (18–21). Physical activity is defined as “any bodily movement produced by skeletal muscles that results in energy expenditure. The energy expenditure can be measured in kilocalories. Physical activity in daily life can be categorized into occupational, sports, conditioning, household, or other activities”. (p 124, 22). A recent survey carried out by ONAPS^1^ in 2023, shows that students from health, science and engineering backgrounds are significantly more sedentary compared with other courses. They indicate that the absence of a free teaching unit devoted to physical activity is a major obstacle. In addition, it has been shown that physically active students are more emotionally competent (23–25). Or, some studies integrate physical activity as a tool to develop EC (26, 27). However, there are few studies based on physical activity to determine whether this tool could be relevant to the development of EC. The aim of this study is to observe whether physical activity can be a relevant tool for developing the emotional skills of students in health training.

## Method

### Participants

68 students in health training participated in a pilot study. To participate, students had to be enrolled in the third year of a bachelor's degree in Sports Science (STAPS) with a specialization in Adapted Physical Activities and Health (APAS) and who did not engage in physical activity outside of their academic studies. Our choice of population focused on these students because they no longer have integrated sports practice in their curriculum in the third year. The choice to include participants based on voluntary participation seemed important to us in order to prevent dropouts during the program. The gender distribution is not provided because several participants chose not to share this information. The majority of students were between 18 and 24 years old, i.e., more than 90% of the sample (*n* = 64). The other participants were in the 25–34 age group (*n* = 4). The age range of membership was preferred to the actual age in order to respect personal data (Statement #123).

Students whose overall global EI level was higher than the top quarter percentile of the sample were excluded from the study.

### Questionnaire

The French version of the TEIque was used in this research (28). The questionnaire consists of 153 items and measures on a total of 13 facets (i.e., self-esteem, emotional expression, emotional regulation, happiness, empathy, social skills, impulsivity, emotional perception, stress management, emotion management, optimism, interpersonal skills, and self-confidence), 2 auxiliary facets (i.e., adaptability and self-motivation), and the overall EI level. The 13 facets allow the scoring of 4 factors: well-being (i.e., self-esteem, happiness and optimism items); self-control (i.e., emotional regulation, impulsivity and stress management items); emotional skills (i.e., emotion perception, empathy and emotion management items) and sociability (i.e., social competence, assertiveness and interpersonal skills items). The participants had to rate these items on a scale of 1 (= completely disagree) to 7 (= completely agree). The internal consistency of the French validation of global trait EI has a Cronbach's alpha of .95.

### Procedure

A presentation of the research training was made in the training institutions in order to recruit student volunteers to participate in the interventions. An email is then sent to interested students to inform them about the training organization. They are informed beforehand by the scientific manager of the study objectives, its methodology, its duration, its constraints and foreseeable risks. Participants are specially informed that they are entirely free to refuse to participate to the study and to withdraw their consent at any time without incurring any liability or prejudice as a result. They are also informed that they can ask their data destruction. A consent form is sent to participants for signature before the study starts. Students had the choice of not participating in the study, participating as part of the control group, participating as part of the experimental-practice group. Two groups are formed: a control group that received an intervention on a topic other than emotional intelligence (i.e., sociological approach of the body) during 18 h (*n* = 34) and one intervention group on emotional intelligence: experimental-practice (*n* = 34) The TEIque was completed electronic format or paper format when it was not possible to complete the questionnaire electronically by all participants: before starting the program and at the end of the program to assess the effectiveness of the intervention programs. A declaration to the Ethic Committee for the Research in sports sciences students (CERSTAPS) and to the CNIL was made in the context of this thesis and therefore for this study. The principle of data's anonymization is guaranteed to the participants.

### Intervention

The intervention training was implemented between October 2020 and October 2021. EC intervention training was designed for this research. The experimental-practice training consisted of theoretical content on EI and practical work. This training involved 34 students in health students participating in a program combining 8 h of theory and 10 h of practical exercises. The theoretical content aims to provide students with knowledge about emotional competencies and their relevance, particularly in the context of healthcare professions. It is based on scientific literature (e.g., 29) and serves to introduce the practical sessions related to physical activity. The practical exercises consist of engaging in playful activities designed to get students moving and focused on the five major emotional competencies based on scientific literature (i.e., 26, 27, 30–32). The duration of the intervention program was set at 18 h following the study by Nelis et al. (29), which showed that an 18 h program was sufficient to improve emotional skills. The introductory session of the program aimed to conduct the first administration of the TEIQue, present the program, and provide an intervention on Emotional Intelligence (EI) in health education. The following three sessions were organized as follows: a theoretical session to provide knowledge on one or more emotional competencies, followed by one or two practical sessions in the gymnasium related to the emotional competencies covered in the theoretical content (Table 1).

**Table 1:** Content EC program.

Topic		Time
Introduction session		2h
Tools	Objectives	2h
Conference	Presentation of the program Passing the TEIQue Knowledge of EI and its relevance to health training	
Session 1 - Expression and understanding of emotions		4h
Conference	Theoretical knowledge on the skills of expressing and understanding emotions - Identifying what happens to us when we listen to music representing particular emotions (e.g., joy, fear, sadness) - Exercises in expressing emotions (mime, dance) - Debriefing of emotional situations	2h
Practice exercices	Physical exercises to learn to express and understand emotions - Exercise to stimulate non-verbal communication - Identification of images related to emotional expressions	2h
Session 2 - Identification of other people's emotions and regulation of emotions		6h
Conference	Theoretical knowledge on the skills of identifying other people's emotions and regulating one's own emotions	2h
Practice exercices	Performing physical exercises to learn to identify and understand other people's emotions	2h
Practice exercices	Physical exercises to learn to regulate stress and emotions - Breathing work through a cardiac coherence exercise - Self-massage and stretching of the trapezius and neck muscles - Exercises to put the participants under pressure	2h
Session 3 - Using your emotions, listening to and regulating the emotions of others		6h
Conference	Providing theoretical knowledge on the skills of using one's emotions, listening and regulating the emotions of others	2h
Practice exercices	Physical exercises to learn to use emotions - Exercises to stimulate students’ adaptive and communicative skills through group games	2h
Practice exercices	Exercises to learn how to regulate the emotions of others - Self-defence - Breathing exercises	2h

#### Data analysis

Independent t test, repeated measures analyses of variance and paired sample test were performed for each dimension of the TEIque to compare scores between time 1 and time 2 using JASP software.

## Results

Independent *t*-tests showed that there were no baseline differences between the experimental and the control group on any of the variables under consideration (Table 2).

**Table 2:** Means, standard deviations and significance of differences between experimental and control group prior to EI intervention.

	Control (*n* = 34)	Experimental (*n* = 34)			
Variable	Pre test M (SD)	Pre test M (SD)	*t*	*p*	*d*
SE	4.545 (0.985)	4.690 (0.897)	0.634	0.529	0.154
EXP	3.487 (1.157)	4.157 (1.306)	2.241	0.028	0.544
M	4.774 (0.657)	4.992 (0.794)	0.422	0.674	−0.102
ER	4.419 (0.809)	4.566 (0.971)	0.674	0.502	0.164
HAP	5.228 (0.794)	5.279 (0.737)	0.275	0.784	0.067
EMP	5.147 (0.716)	5.242 (0.646)	0.573	0.569	0.139
SK	4.789 (1.004)	4.725 (0.710)	0.301	0.764	−0.073
IMP	4.686 (0.952)	4.520 (0.697)	0.823	0.413	−0.200
EP	4.756 (0.870)	4.779 (0.712)	0.122	0.903	0.030
SM	4.265 (1.085)	4.647 (1.347)	1.289	0.202	0.313
EM	4.611 (1.057)	4.546 (0.725)	0.297	0.767	−0.072
O	4.820 (1.124)	4.816 (0.948)	0.013	0.990	−0.003
IS	5.667 (0.693)	5.539 (0.640)	0.787	0.434	−0.191
A	4.641 (0.666)	4.575 (0.849)	0.356	0.723	−0.086
ASS	4.503 (0.819)	4.523 (0.608)	0.116	0.908	0.028
WB	4.864 (0.870)	4.928 (0.722)	0.334	0.740	0.081
SC	4.457 (0.761)	4.578 (0.776)	0.647	0.520	0.157
EA	4.500 (0.594)	4.681 (0.487)	1.376	0.173	0.334
S	4.986 (0.606)	4.929 (0.468)	0.441	0.661	−0.107
EI	4.689 (0.579)	4.753 (0.444)	0.516	0.607	0.125

SE, self esteem; EXP, emotional expression; M, motivation; ER, emotional reaction; HAP, happiness; EMP, empathy; SK, social skills; IMP, impulsivity; EP, emotional perception; SM, stress management; EM, emotional management; O, optimism; IS, interpersonal skills; A, adaptability; ASS, assurance; WB, well-being; SC, self-control; EA, emotional abilities; S, sociability; EI, emotional intelligence.

Mixed model group (experimental vs. control) × time (time 1, time 2) repeated measures ANOVAs were performed on each measure, with group as between-subject factor and time as within-subject factor. In each case, we were looking for a group × time interaction, which would indicate a differential change for the two groups.

**Figure 1:**
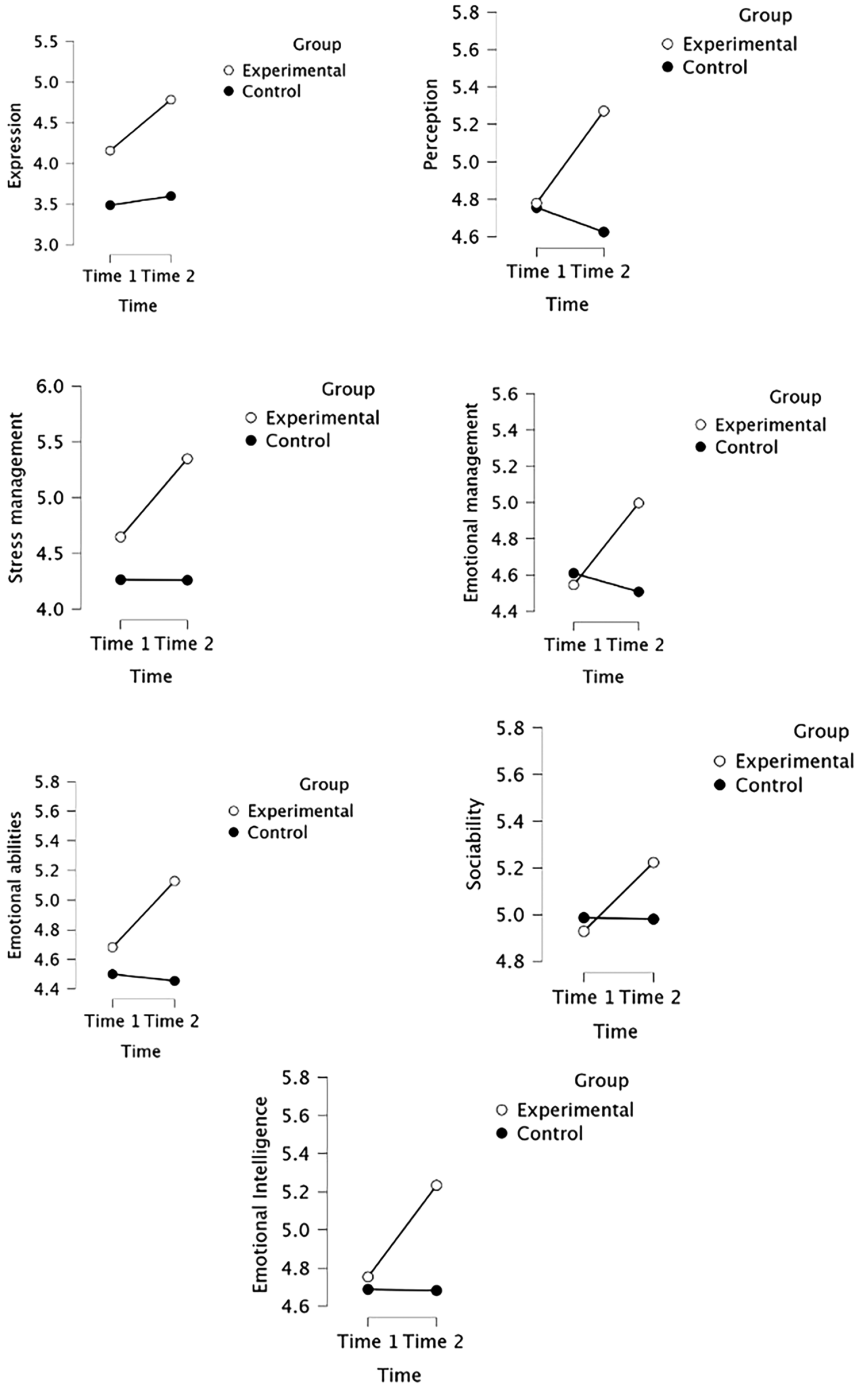
Effect of EI intervention across two time (time 1 = before training, time 2 = after training) of evaluation for the two groups (experimental and control group).

Analyses yielded a significant group × time interaction for expression of emotion [*F* (1,66) = 9.013, *p* = 0.004 and *η*^2^*p* = 0.120]; perception of emotion [*F* (1,66) = 25.013, *p* < .001 and *η*^2^*p* = 0.275]; stress management [*F* (1,66) = 12.790, *p* < .001 and *η*^2^*p* = 0.162]; emotional management [*F* (1,66) = 12.790, *p* < .001 and *η*^2^*p* = 0.151]; emotional abilities [*F* (1,66) = 12.790, *p* < .001 and *η*^2^*p* = 0.322]; sociability [*F* (1,66) = 12.790, *p* < .001 and *η*^2^*p* = 0.160] and emotional Intelligence [*F* (1,66) = 27.871, *p* < .001 and *η*^2^*p* = 0.297]. As illustrated by the descriptive plots for these variables, all effects indicate an increase from pre to pos*t*-test scores in the intervention group when compared to the control group (Figure 1).

The t-statistics related to the comparison of the pre and post test of the different groups, as well as the significant differences after the interventions, are shown in Table 3.

**Table 3:** Student's paired sample and significance of differences after intervention for each variable and each group.

	Control			Experimental		
	Diff	*t*	*p*	Diff	*t*	*p*
SE	−0.019	0.277	0.784	−0.295	2.711	0.011
EXP	−0.111	1.108	0.276	−0.626	4.519	**<.001** **
M	−0.057	0.716	0.479	−0.295	3.056	**0**.004*
ER	0.009	0.103	0.918	0.026	0.150	0.881
HAP	0.029	0.360	0.721	0.022	0.165	0.870
EMP	0.059	0.739	0.465	−0.043	0.304	0.763
SK	−0.002	0.039	0.969	−0.325	3.076	**0**.004*
IMP	−0.014	0.175	0.862	−0.039	0.237	0.814
EP	0.130	1.406	0.169	−0.492	5.922	**<.001** **
SM	0.005	0.043	0.966	−0.701	4.169	**<.001** **
EM	0.103	0.995	0.327	−0.452	3.611	**<.001** **
O	−0.114	1.527	0.136	−0.514	3.403	**0**.002*
IS	0.135	2.581	0.014	−0.186	1.545	0.132
A	0.011	0.144	0.886	−0.043	0.264	0.793
ASS	−0.072	0.834	0.410	−0.032	0.256	0.799
WB	−0.101	1.616	0.116	−0.303	3.153	**0**.003*
SC	−0.013	0.145	0.886	−0.239	1.987	0.055
EA	0.045	0.805	0.427	−0.446	6.631	**<.001** **
S	0.005	0.139	0.890	−0.295	4.018	**<.001** **
EI	0.006	0.178	0.860	−0.480	5.667	**<.001** **

Values in bold correspond to significant values.

* *p* < .005.

** *p* < .001.

EP group showed a significant increase on global EC, *d* = 0.97; Sociability, *d* = 0.69; Emotional Abilities, *d* = 1.14; Well-being, *d* = 0.54; Optimism, *d* = 0.58; Emotional Management, *d* = 0.62; Stress Management, *d* = 0.72; Emotional Perception, *d* = 1.016; Social skills, *d* = 0.53; Motivation, *d* = 0.52 and Emotional Expression, *d* = 0.78. Conference group showed a significant increase on global EC, *d* = 0.80; Emotional Perception, *d* = 0.58; Stress Management, *d* = 0.71; Adaptability, *d* = 0.71; Interpersonal Skills, *d* = 0.78 and Optimism, *d* = 0.83. As expected, the control group showed no significant difference between pre and post test on most measures (*p* ranging from 0.014 to 0.969).

## Discussion

The purpose of this study was to examine whether a theoretical and practical training program on EC could increase the EC level of students in health education. The results obtained allow us to highlight several effects of our intervention program.

We observe significant differences after the intervention for the participants in the intervention program and no statistical difference for the control group. The results of the experimental group show an improvement in global EC, emotional expression, emotional perception, stress management, emotional management, social skills, sociability, motivation, optimism, emotional abilities and well-being. The program offered to the students consisted of theoretical conferences on different EC and then the application of the theoretical content in practical exercises. Among the exercises, several targeted expressing, identifying and regulating one's own emotions and those of others. These exercises, for the most part, derived from the work conducted by Laborde et al. (27) and Fernandez-Gamez (26), have been proven to improve these different skills. Our results thus confirm the previous results obtained by these authors. In addition, other activities such as self-defense or dance were integrated into our practical sessions. The self-defense session aimed providing the students with a basis to learn how to communicate and to manage conflict and emotions. Indeed, a previous study showed that intervention programs directed towards these activities improved cognitive and emotional self-regulation skills and social behavior of children (31). In addition, another study conducted on 120 subjects practiced a combat sport (i.e., judo or boxing) and non-athletes showed that the practitioners obtained higher scores on perception, use and control of emotions. The dance session purpose was to express and understand the emotions felt in the activity. A review of the literature on emotional intelligence and dance showed that this activity was beneficial for the emotional and affective development of the individual (30). This justifies the choice of this activity. In addition, cardiac coherence exercises for breathing and stretching exercises and self-massage for muscle relaxation were also implemented to teach students to better manage their stress and relax. The improvement in stress management skills of the participating students suggests that these activities were effective. Moreover, a study conducted by Sarabia-Cobon (33) obtained similar results during its cardiac coherence program conducted with 72 careers. Furthermore, the aim of the practical sessions was to set up playful exercises that promote cooperation and communication between the participants. This could explain the improvement in the level of sociability of this group. Our results confirm those of previous works which showed that a cooperative dynamic increases motivation and improves self-efficacy and pro-social behavior (34–36). Regarding the improvement of well-being, it could be associated with the practice of exercises around physical activities within the practical sessions. Indeed, studies show the influence of activity on the level of well-being of students (23, 37).

Moreover, contrary to what one might have thought, there was no improvement in the level of empathy for all groups. There are two reasons for this. Firstly, analyzing the pre-test results conducted to see that the empathy level was above 5 for the groups. This could be explained by the fact that the level of empathy could be a characteristic of students choosing to go into health studies. Indeed, a study of 363 medical students found that high levels of empathy were associated to choose a person-centered rather than technology-centered medical career (38). It would be interesting to conduct a study with students to confirm this hypothesis and thus observe whether certain personality traits influence the choice of career orientation.

## Conclusion

The implementation of an intervention program based on the tripartite model of emotional intelligence aimed to evaluate a pedagogical modality coupling theory and practice. The aim was to establish a recommendation for integrating a free teaching unit on physical activity and emotional competencies in health training. These results are encouraging regarding the use of the tripartite model to address the concept of EC. They provide interesting perspectives on its use in health training. The results obtained are even more meaningful in the light of the health crisis we experienced. Indeed, epidemic COVID-19 was accompanied by an increase in anxiety and depressive disorders, sleep disorders, acute stress disorders, and post-traumatic stress disorders among healthcare workers (39, 40). The increase in these disorders showed that the health workers and students involved in the crisis were not prepared to face this kind of situation (41). Our study represents an interesting lever for learning to better manage similar situations in the future. However, EC oriented care could act on all three stages of work-related stress prevention. Indeed, for primary prevention, this early management would prepare students to cope with difficult situations through the integration of emotionally intelligent behaviors such as reflection and reframing, compromise, calmness, control of discomfort and appropriate expression of emotions (42, 43). Secondary prevention can be carried out with students or nurses who are aware that they have difficulties managing their stress or expressing their emotions in a way that limits the consequences that could be harmful to their health. To do this, discussion groups, breathing exercises, physical activity or tools that encourage the expression of emotions can be used (25, 33, 44, 45). EI-oriented interventions can also be used in tertiary prevention for the management of post-traumatic stress or burnout (46, 47). Our study also provides an interesting path for future research to be extended to all types of audiences who have experienced difficult emotional situations.

However, our study has some limitations. Regarding the sample size, this was a pilot study based on voluntary participation. Therefore, the size of our sample depended on the students' willingness to join the intervention program. Some students expressed that they did not feel the need for interventions to learn how to manage or express their emotions. Other researchers have taken a different approach. For example, Campo et al. (48) included an entire rugby team in his intervention on Emotional Intelligence (EI) without considering the athletes' interest in this type of practice. Following this program, he observed a significant improvement in EI, even among athletes who had not shown particular motivation to engage in this type of exercise (48). Such interventions could, therefore, be beneficial for everyone, even if individuals do not initially express a specific need for them. A larger sample would have provided a more representative cross-section of this student population. Moreover, unlike some studies, we did not take the variable gender to account EI level. We made this choice for several reasons. The gender distribution is not provided because several participants chose not to share this information. Furthermore, a large proportion of students in health training programs are female. In this context, it seemed difficult to obtain representative data given the gender disparity in our sample, especially since our sample was not randomized but constructed based on voluntary participation. It would be interesting to take this variable into account in the future to verify whether, as some studies have shown, gender is a significant moderator of the relationship between medical students' emotional intelligence and stress coping (25, 49). Regarding the intervention program, we only evaluated the effectiveness of a program combining theory and practice for the development of emotional competencies. Therefore, we cannot determine whether it was the practical exercises, the theory, or the theory/practice program that contributed to improving the students' emotional competencies. It would have been interesting to have an intervention group that completed 18 h of theory only and another that did 18 h of practical exercises only, in order to determine the most effective modality. These elements thus provide us with interesting avenues to explore in the context of future research.

## Data Availability

The original contributions presented in the study are included in the article/Supplementary Material, further inquiries can be directed to the corresponding author.

## Footnotes

^1^ONAPS. (2023). Pratique d’activités physiques et sportives et la sédentarité chez les étudiants en formation universitaire en France, 1-34. https://onaps.fr/enquete-sur-la-pratique-dactivites-physiques-et-sportives-et-la-sedentarite-a-luniversite/
